# Kentucky Dental Association

**Published:** 1872-08

**Authors:** 


### Kentucky Dental Association.

The Kentucky State Dental Association was held at Louisville on Tuesday, June 4, 1872. The association convened at 2 o'clock P. M., at the office of Dr. J. F. Canine, and was duly organized, then adjourned until Wednesday morning at 9 o'clock. The mornings from 9 to 12 were devoted to clinics.

SECOND DAY'S PROCEEDINGS.

The Association met at 9 o'clock at the appointed place to witness the clinic by Drs. Goddard and Canine. Dr. Goddard operated on a second bi-cuspid which was witnessed by those present with great interest. Dr. Canine operated on a lateral incisor, which was also witnessed with interest by all present.

These clinics are practically of more value than any other part of the proceedings. The clinic to-morrow will be by Dr. J. Richardson, of Terre Haute, Ind., and Drs. Redman and Peabody.

AFTERNOON SESSION.

The special order for this hour was the election of officers, which resulted as follows:

President—Dr. W. H. Goddard.

Vice President—Dr. C. E. Dunn.

Secretary—Dr. G. W. Priest.

Treasurer—Dr. J. F. Canine.

Members Board of Censors—Drs. Redman, Peabody and Dunn.

To fill vacancy in Executive Committee—Drs. Redman and Priest.

The subject of exposed pulps of the teeth was then taken up and discussed by Drs. Peabody, Redman, Richardson and Priest. The subject was ably discussed and brought out several very excellent ideas.

THIRD DAY.

Morning clinics by Dr. J. Richardson, of Terre Haute, Dr. F. Peabody, and, by special request, Dr. Seymour, who filled a very frail and difficult cavity in the first left superior bi-cuspid, showing to those present this manner of filling and building a large contour, filling with needle pointed instruments and hand pressure, using soft gold, which was very interesting, and gave great pleasure and satisfaction to all.

The association was called to order by the President at o'clock P. M.

Reading of the minutes was dispensed with. Business in the regular order was taken up. No reports from committees. Miscellaneous business being in order, the names of Drs. B. Oscar Doyle, of Louisville; H. Baldwin, of Elizabethtown; J. P. Gillespie, of Louisville; R. C. Morgan, D. D. S., of Columbia, and L. W. Wells, of Paducah, were proposed for membership and referred to the Board of Censors. The following members of the Executive Committee, Drs. Canine and Dunn, in connection with Dr. Priest, the Recording Secretary, were appointed as a publication committee. The election of delegates to the American Dental Assciation being in order, Drs. Priest and Redman were unanimously elected. Drs. C. E. Dunn and F. Peabody were elected delegates to the Southern Dental Association.

The association then took up for discussion—"filling teeth and material for the same." The discussion was opened by Dr. J. Richardson, of Terre Haute, Ind., explaining in a very lucid manner the preparation of the various cavities preparatory to filling. The discussion was continued by Drs. Redman, Canine, Shadoan, Morgan and others, showing the necessity of devoting more time and attention to that part of the operation. The discussion on filling teeth and preparation of cavities was closed. Dr. Shadoan made a protracted speech on the use of rubber dam and the manner of applying it. Dr. Redman thought too much importance was attached to the use of rubber dam, and especially too much valuable time was consumed in its application. Dr. Canine agreed with Dr. Redman, stating that he had operated for persons who would not submit to its application by other dentists He had used rubber dam whenever he thought it necessary.

Dr. Peabody thought its use was very important; indeed almost indispensable in many cases, and thought a familiarity with its use would make it a more welcome adjunct to the profession.

Dr. Wells, of Paducah, had much difficulty in its application, and for a time abandoned its use.

Dr. Prevost thought that it was seldom necessary to use it in cylinder fillings; that in the use of adhesive foil, that or something else was indispensable to protect the gold in process of filling from moisture.

Dr. Gillespie thought rubber dam one of the most valuable articles in the dental profession. All present, however, agreed that it was indispensable in many cases.

On motion, the subject of rubber dam was closed.

The association then adjourned to meet at eight o'clock P. M.

EVENING SESSION.

The association met according to adjournment. On motion, the remaining subjects for discussion were embraced under one head.

A general conversation upon irregularities was participated in by all present, and various plans were suggested for accomplishing the same object; and various models of irregularity were presented by Drs. Richardson and Canine and their methods for regulating the same explained. The subject of sensitive dentine was fully discussed, and all the various remedies usually tried were found not to answer the object to be obtained, except sharp excavators, with quick and heroic preparation of the cavity. In the rapid advancement the profession is now making we will soon find some remedy by which we can in a great measure, if not fully, relieve the patients of their suffering.

Drs. Richardson, Morgan, Wells and Gillispie, each expressed his great gratification in being present at this meeting, thanking the association for the invitation, and stating that they had enjoyed it and greatly profited by its discussions, and hoped to be present again.

On motion of Dr. Wells, a vote of thanks was tendered the officers and members of the association for the courtesy and kindness extended to the visiting brethren.

The President responded to the courteous remarks and resolutions of thanks from the visiting brethren in a feeling and touching manner, extending to them, in behalf of the association, a cordial welcome to any and all of the meetings when in the city.

The members of the association and the visiting brothers were invited to meet for social purposes on Tuesday evening, at the office of Dr. Canine. Many pleasant thoughts were exchanged and discussed; also other refreshing matters of a more substantial nature. Wednesday evening Dr. Goddard invited all to his house, where many laughable experiences and anecdotes were related, and the inner man agreeably refreshed. The evenings were pleasantly passed and enjoyed by all. A number of appliances and improvements in dentistry were exhibited. Among them were the "new atmospheric attachments to dental plates," by Dr. J. P. Gillispie, and his "diamond pointed drills," both of which appear to be valuable improvements in dentistry.

A vote of thanks was tendered to Dr. Canine for the use of his elegant parlors for the meetings of the association.

On motion, the association adjourned.

